# Gamma-Irradiated Bacille Calmette-Guérin Vaccination Does Not Modulate the Innate Immune Response during Experimental Human Endotoxemia in Adult Males

**DOI:** 10.1155/2015/261864

**Published:** 2015-03-26

**Authors:** Linda A. C. Hamers, Matthijs Kox, Rob J. W. Arts, Bastiaan Blok, Jenneke Leentjens, Mihai G. Netea, Peter Pickkers

**Affiliations:** ^1^Department of Intensive Care Medicine, Radboud University Medical Center, Radboud Center for Infectious Diseases (RCI), P.O. Box 9101, 6500 HB Nijmegen, Netherlands; ^2^Department of Internal Medicine, Radboud University Medical Center, Radboud Center for Infectious Diseases (RCI), P.O. Box 9101, 6500 HB Nijmegen, Netherlands

## Abstract

Bacille Calmette-Guérin (BCG) vaccine exerts nonspecific immunostimulatory effects and may therefore represent a novel therapeutic option to treat sepsis-induced immunoparalysis. We investigated whether BCG vaccination modulates the systemic innate immune response in humans *in vivo* during experimental endotoxemia. We used inactivated gamma-irradiated BCG vaccine because of the potential risk of disseminated disease with the live vaccine in immunoparalyzed patients. In a randomized double-blind placebo-controlled study, healthy male volunteers were vaccinated with gamma-irradiated BCG (*n* = 10) or placebo (*n* = 10) and received 1 ng/kg lipopolysaccharide (LPS) intravenously on day 5 after vaccination to assess the *in vivo* immune response. Peripheral blood mononuclear cells were stimulated with various related and unrelated pathogens 5, 8 to 10, and 25 to 35 days after vaccination to assess *ex vivo* immune responses. BCG vaccination resulted in a scar in 90% of vaccinated subjects. LPS administration elicited a profound systemic immune response, characterized by increased levels of pro- and anti-inflammatory cytokines, hemodynamic changes, and flu-like symptoms. However, BCG modulated neither this *in vivo* immune response, nor *ex vivo* leukocyte responses at any time point. In conclusion, gamma-irradiated BCG is unlikely to represent an effective treatment option to restore immunocompetence in patients with sepsis-induced immunoparalysis. This trial is registered with NCT02085590.

## 1. Introduction

Sepsis is a clinical condition that represents a major medical challenge due to its high mortality rate. Related to this, it is a major clinical challenge also because it may be difficult to diagnose in due time and difficult to treat. Previous adjunctive therapeutic strategies, aiming to treat sepsis by inhibition of proinflammatory mediators, have failed, likely related to the recent insight that the majority of septic patients do not succumb to the initial proinflammatory “hit” but die at a later time point in a pronounced immunosuppressive state [1–3]. This so-called “sepsis-induced immunoparalysis” results from counterregulatory anti-inflammatory pathways that are activated simultaneously with proinflammatory mechanisms [2–4]. This renders patients unable to clear the initial infection and increases vulnerability to secondary infections [2, 3, 5]. As a consequence, reconstitution of immunocompetence is emerging as a new and promising therapeutic target to improve outcome in sepsis patients [2, 3, 6, 7].

Bacille Calmette-Guérin (BCG) is the most widely used vaccine worldwide. In addition to protection against tuberculosis [8], both observational studies and randomized clinical trials have shown that BCG vaccination is associated with beneficial effects on other infectious diseases as well. In this regard, early administration of BCG vaccination leads to strongly reduced infant mortality, mainly as a result of lower incidence of neonatal sepsis, respiratory infection, and fever [9–15]. These nonspecific effects of BCG are suggested to be mediated by two mechanisms: potentiation of adaptive immune responses against unrelated pathogens, the so-called “heterologous immunity” [16], and epigenetic functional reprogramming of innate immune cells to an enhanced phenotype, a process described as “trained immunity” [17–20]. The latter process is characterized by enhanced production of proinflammatory cytokines by monocytes that have been previously primed by BCG and is apparent upon stimulation with either specific or unrelated pathogens [17, 21]. In line with this, mononuclear cells isolated from healthy volunteers and stimulated with unrelated pathogens show enhanced innate immune responses after BCG vaccination [17], and some of these effects even persist for up to one year [21]. These effects largely rely on innate immune cells, as BCG vaccination enhances resistance against* Candida* infection and increased lipopolysaccharide- (LPS-) induced cytokine production in splenocytes of mice lacking T- and B-cells [17].

Considering its potentiating effects on host defense, BCG could represent a therapeutic option to prevent or treat sepsis-induced immunoparalysis. Nevertheless, as patients with sepsis have an increased susceptibility to secondary infections, vaccination with live BCG may be associated with unwarranted risks for dissemination [22]. As recent data showed that gamma-irradiated BCG has similar potentiating effects on trained immunity* in vitro* (Arts et al., submitted) but does not present any risk for infection, this inactivated form of BCG represents a clinically relevant alternative in these patients. However, the effects of BCG vaccination on the immune response in humans have hitherto only been shown* ex vivo* [17, 21]. It has yet to be established whether these findings can be extrapolated to the human* in vivo* situation, because* ex vivo* data might not always reflect* in vivo* responses [23, 24]. The human endotoxemia model, in which a low dose of LPS is administered to healthy volunteers, represents a unique model to study modulation of the systemic inflammation in humans* in vivo* in a safe, highly standardized, and reproducible manner [25].

The aim of the present study was to investigate the effects of vaccination with gamma-irradiated BCG on the systemic innate immune response in adult males* in vivo* during experimental endotoxemia.

## 2. Methods

### 2.1. Subjects

After approval from the Arnhem-Nijmegen Ethics Committee, 20 healthy nonsmoking male volunteers gave written informed consent to participate in this study that was registered at ClinicalTrials.gov as NCT02085590. Subjects were screened before the start of the experiment and had a normal physical examination, electrocardiography, and routine laboratory values. Exclusion criteria were febrile illness during the 2 weeks before start of the study, prior BCG vaccination, any vaccination other than BCG within 3 months before start of the study, and a tuberculin skin test within 1 year prior to the start of the study. Throughout the study period, subjects were not allowed to take any drugs, including acetaminophen, and were asked to refrain from alcohol and caffeine 24 hours and from food 12 hours before the start of the endotoxemia experiment. All study procedures were conducted in accordance with the declaration of Helsinki including current revisions and Good Clinical Practice guidelines.

### 2.2. Study Design and Procedures

**Figure 1:**
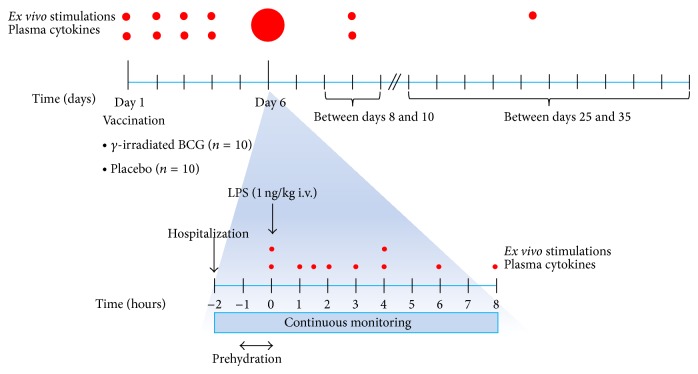
Experimental design. BCG: Bacille Calmette-Guérin; LPS: lipopolysaccharide.

We performed a randomized double-blind placebo-controlled study. The study design is schematically depicted in Figure 1. For reasons detailed in the Introduction, gamma-irradiated (and therefore inactivated) BCG vaccine was used in this study. Irradiated BCG was cultured for 6 weeks using Mycobacteria Growth Indicator Tubes according to Dutch national guidelines to confirm inactivation, and no growth was observed. Subjects were randomly assigned to receive either 0.075 mg (0.1 mL) gamma-irradiated BCG vaccine intracutaneously (BCG vaccine SSI; Statens Serum Institut, gamma-irradiation (25–30 kGy) performed by Synergy Health Ede, Netherlands; *n* = 10) or 0.1 mL placebo (BCG-reconstitution fluid: diluted Sauton 1+3; Statens Serum Institut; *n* = 10) in a double-blind fashion. Five days after vaccination, all subjects received an intravenous injection of LPS (lipopolysaccharide derived from* Escherichia coli* O:113, Clinical Center Reference Endotoxin, National Institutes of Health (NIH), Bethesda, MD), 1 ng/kg. Endotoxemia experiments were conducted as described previously [24]. Heart rate (three-lead electrocardiogram), blood pressure, respiratory rate, and oxygen saturation (pulse oximetry) data were recorded from a Philips MP50 patient monitor every 30 seconds by a custom in-house-developed data recording system. LPS-induced flu-like symptoms (headache, nausea, shivering, muscle, and back pain) were scored every 30 min on a six-point Likert scale (0 = no symptoms, 5 = worst ever experienced), resulting in a total score of 0–25 points. After the endotoxemia experiment, additional blood samples were drawn on days 8–10 and days 25–30 after vaccination. During the last visit, BCG scar formation was measured by an independent research nurse (to maintain blinding) using a centimeter ruler.

### 2.3. Cytokine Measurements

To analyze plasma cytokines, ethylenediaminetetraacetic acid (EDTA) anticoagulated blood was centrifuged at 2,000 ×g at 4°C for 10 minutes immediately after withdrawal, and plasma was stored at −80°C until analysis. Concentrations of TNF-*α*, IL-6, IL-8, IL-10, IL-1*β*, IL-1 receptor antagonist (IL-1ra), MCP-1, and IFN-*γ* were measured in plasma batchwise using a Luminex assay according to the manufacturer's instructions (Milliplex; Millipore, Billerica, MA, USA).

### 2.4. Leukocyte Counts and Differentiation

Analysis of leukocyte counts and differentiation was performed in EDTA anticoagulated blood using routine analysis methods also used for patient samples (flow cytometric analysis on a Sysmex XE-5000).

### 2.5. Peripheral Mononuclear Cell Stimulation Assays

The mononuclear cell fraction was isolated by density centrifugation of EDTA anticoagulated blood, diluted 1 : 1 in pyrogen-free saline, over Ficoll-Paque (Pharmacia Biotech, Uppsala, Sweden). Isolated cells were washed twice in saline and resuspended in culture medium (RPMI, Invitrogen, Carlsbad, California, USA) supplemented with 10 *μ*g/mL gentamicin, 10 mM L-glutamine, and 10 mM pyruvate. Cell counts were performed in a Coulter counter (Coulter Electronics). A total of 5 × 10^5^ mononuclear cells in 100 *μ*L were added to round-bottomed 96-well plates (Greiner) with RPMI, sonicated* Mycobacterium tuberculosis* (MTB) (1 *μ*g/mL end protein concentration, strain H37Rv),* Escherichia coli* lipopolysaccharide (LPS; 1 ng/mL; Sigma-Aldrich, St. Louis, MO, USA), heat-killed* Staphylococcus aureus* (1 × 10^6^ microorganisms/mL, clinical isolate), or heat-killed* Candida albicans* (1 × 10^6^ microorganisms/mL, strain UC820). After 24 h (for determination of TNF-*α*, IL-1*β*, and IL-6) or 48 h (for determination of IFN-*γ* and IL-10) of incubation, plates were centrifuged and supernatants were stored at −20°C until analysis. Cytokines were measured batchwise using commercially available ELISAs (R&D Systems, MN, USA, and Sanquin, Amsterdam, Netherlands) according to the protocols supplied by the manufacturers.

### 2.6. Calculations and Statistical Analysis

Data are represented as median and interquartile range or mean and SEM, based on their distribution (calculated by the Shapiro-Wilk test). The area under the curve (AUC) of cytokine levels during experimental endotoxemia, representing an integrated measure of the cytokine response, was calculated using time points 0–8 hours after LPS. Comparisons were made using Mann-Whitney *U* tests (nonnormally distributed data, between-group comparisons) or repeated measures two-way ANOVA (normally distributed data, where the time factor represents differences across both groups over time and the interaction factor represents between-group differences over time).* Ex vivo* cytokine data were log-transformed to obtain a normal distribution. A *P* value < 0.05 was considered statistically significant. Calculations and statistical analyses were performed using Graphpad Prism version 5.0 (Graphpad Software, San Diego, CA, USA).

## 3. Results

### 3.1. Baseline Characteristics

No differences in baseline characteristics between both groups were present (Table 1). Gamma-irradiated BCG vaccination resulted in a scar at the vaccination site in 9 out 10 subjects (median (range) size of 6 (1–9) mm). In the placebo group, 1 subject developed a small scar (1 mm). BCG vaccination did not result in fever or other clinical symptoms and no serious adverse events occurred during the trial.

### 3.2. Hemodynamic Parameters and Symptoms

**Figure 2:**
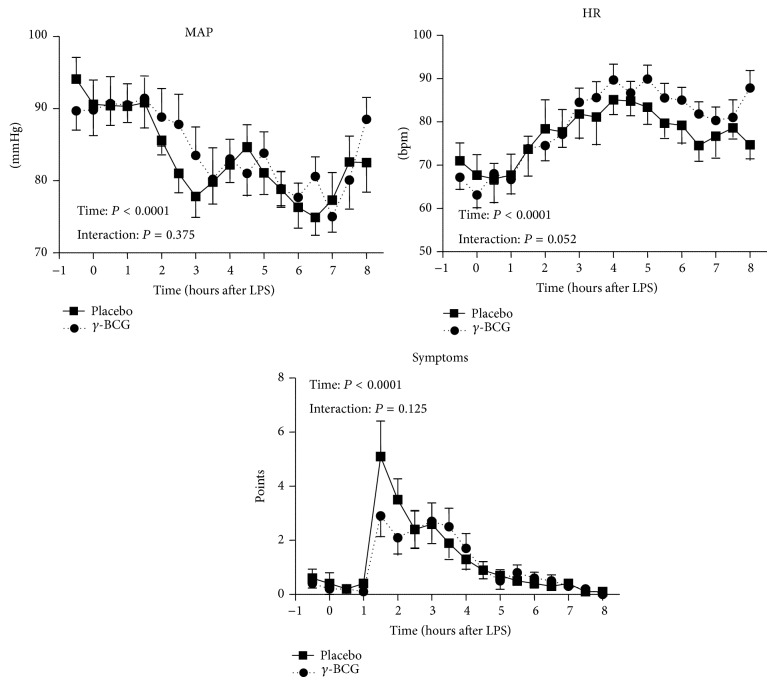
Blood pressure, heart rate, and symptoms during experimental endotoxemia in subjects vaccinated with gamma-irradiated BCG or placebo. Data are expressed as mean ± SEM (*n* = 10 per group). *P* values calculated using repeated measures two-way analysis of variance (ANOVA, time and interaction terms). MAP: mean arterial pressure; HR: heart rate; bpm: beats/min.

LPS administration resulted in a typical increase in heart rate and flu-like symptoms and a decrease in mean arterial blood pressure (MAP) in all subjects, with no differences between groups (Figure 2).

### 3.3. Plasma Cytokines and Circulating Leukocyte Counts

**Figure 3:**
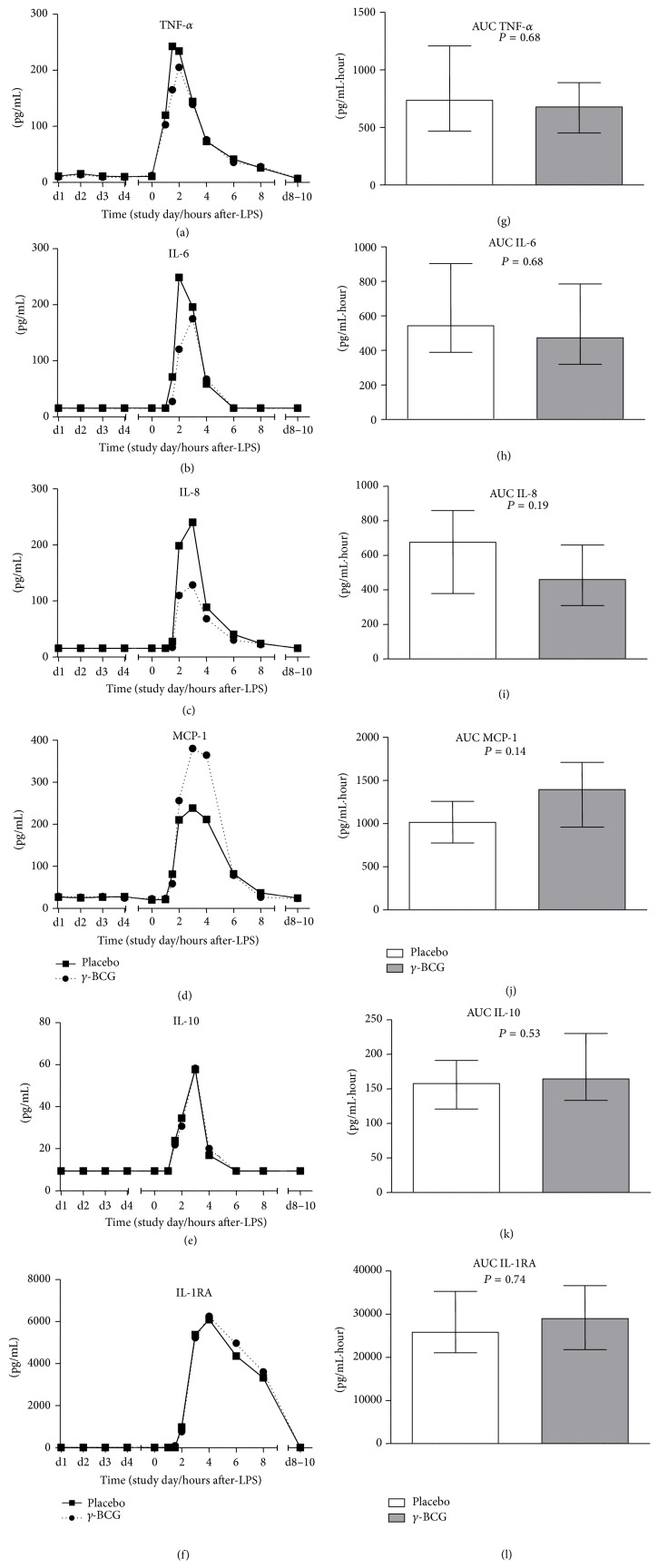
Plasma cytokine concentrations in subjects vaccinated with gamma-irradiated BCG or placebo. In the panels (a–d), median values of proinflammatory cytokines TNF-*α*, IL-6, IL-8, and MCP-1 are depicted while in panels (e) and (f) median values of anti-inflammatory cytokines IL-10 and IL-1RA are shown (*n* = 10 per group). Panels (g–l) depict median ± interquartile range of area under curve (AUC) of the respective cytokines (*n* = 10 per group). *P* values calculated using Mann-Whitney *U* tests.

BCG vaccination did not result in increased plasma levels of any of the measured cytokines in the days following vaccination (Figure 3). As expected, administration of LPS resulted in a sharp increase in plasma levels of the proinflammatory cytokines TNF-*α*, IL-6, IL-8, and MCP-1, as well as the anti-inflammatory cytokines IL-10 and IL-1RA (Figure 3). However, no differences were observed between the BCG and placebo groups. Similar to previous human endotoxemia studies [26], plasma levels of IL-1*β* and IFN-*γ* were below the lower detection limits in the majority of the subjects at most time points. In some subjects and/or time points, very low levels (approximately 10 pg/mL) were found, but no clear patterns over time or differences between groups were observed.

As described earlier, scar size differed substantially between BCG-vaccinated subjects, which might represent vaccination efficacy, and is associated with nonspecific beneficial effects of BCG [27, 28]. Therefore, we stratified the BCG-vaccinated group according to scar size (≤5 mm, *n* = 5; >5 mm, *n* = 5). These stratified analyses did not reveal notable differences in cytokine responses either (see Supplementary Figure 1 in the Supplementary Material available online at http://dx.doi.org/10.1155/2015/261864).

After LPS administration, transient leukocytosis developed, reaching maximum levels at *T* = 8 hours, with no differences between groups (mean ± SEM of BCG and placebo groups, respectively: 10.2 ± 0.7 versus 9.7 ± 0.7 × 10^9^/L, *P* = 0.62). At the first visit after the endotoxemia day (days 8–10), leukocyte numbers were normalized in both groups (5.2 ± 0.4 versus 5.7 ± 0.6 × 10^9^/L in the BCG and placebo groups, respectively, *P* = 0.50).

### 3.4. Cytokine Production by Peripheral Blood Mononuclear Cells

**Figure 4:**
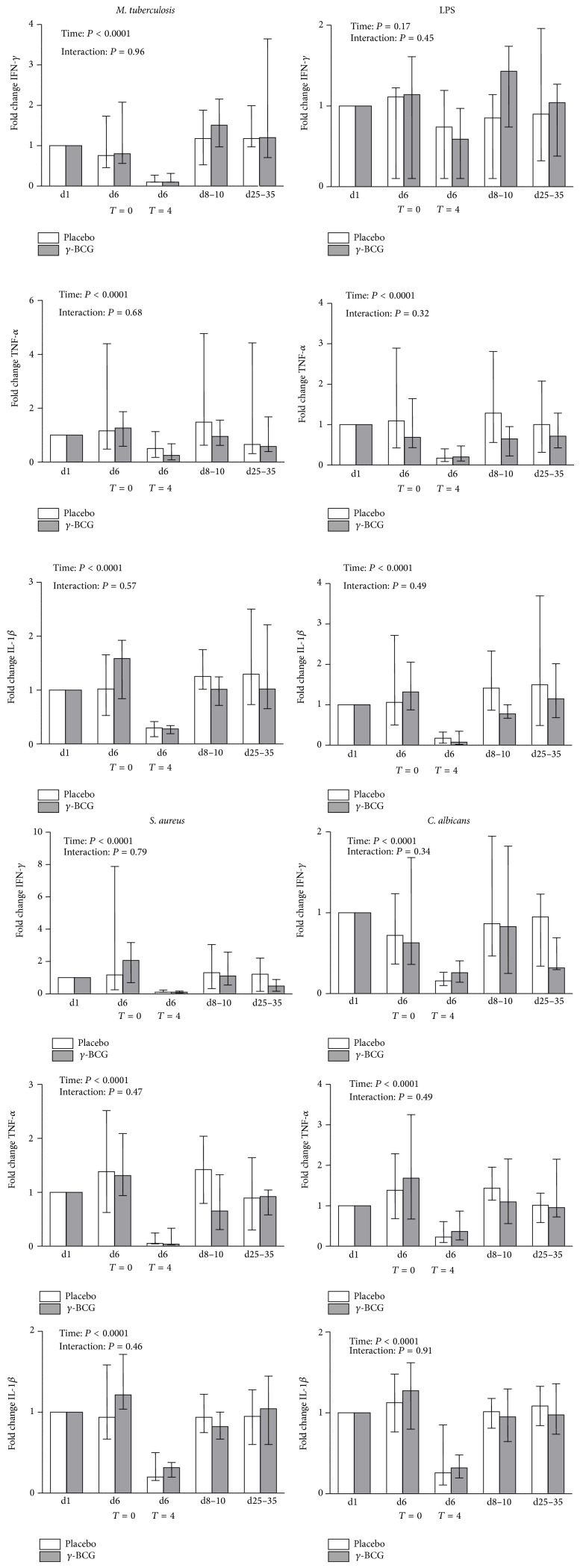
Production of IFN-gamma, TNF-*α*, and IL-1*β* by peripheral blood mononuclear cells stimulated* ex vivo* with* Mycobacterium tuberculosis *(MTB), LPS,* Staphylococcus aureus *(SA), and* Candida albicans *(CA) of subjects vaccinated with gamma-irradiated BCG or placebo. Data expressed as median and interquartile range of the fold change compared with day 1 (before vaccination) (*n* = 10 per group). *P* values calculated using repeated measures two-way analysis of variance (ANOVA, time and interaction terms) on log-transformed data. Day 6 was the endotoxemia experiment day.

Five days after vaccination with BCG or placebo but before LPS administration* in vivo*, there were no differences between groups in* ex vivo* cytokine responses induced by specific (*Mycobacterium tuberculosis*) or unrelated (LPS,* Staphylococcus aureus*, and* Candida albicans*) pathogens or stimuli (fold change data (compared with baseline) of IFN-*γ*, TNF-*α*, and IL-1*β* are depicted in Figure 4, and fold change data of IL-6 and IL-10 are in Supplementary Figure 2. Absolute values of all cytokines are depicted in Supplementary Figure 3). Similar to previous endotoxemia experiments [24, 29], four hours after LPS administration, an overall profound decrease in* ex vivo* cytokine production was observed, indicative of immunoparalysis. BCG vaccination did not influence the development or magnitude of immunoparalysis. Likewise, no differences between groups in* ex vivo* cytokine responses to any of the pathogens or stimuli were found on days 8–10 and 25–35 after vaccination. Of note, LPS-induced production of IFN-*γ*, as well as LPS- and* Mycobacterium tuberculosis-*induced production of IL-10, was absent in many subjects and very low in others. Therefore, the endotoxemia-induced decrease in* ex vivo* cytokine production was less noticeable and did not always reach statistical significance for these combinations.* Staphylococcus aureus-* and* Candida albicans*-induced IL-10 production was absent in virtually all subjects and was therefore not analyzed.

## 4. Discussion

In the present study, we demonstrate that gamma-irradiated BCG vaccination does not influence the LPS-induced innate immune response in adult males* in vivo* five days later. Furthermore, no effects of BCG vaccination on cytokine production of leukocytes stimulated* ex vivo* with specific and unrelated pathogens were observed.

As all measured parameters were similar between groups, we can conclude that five days after vaccination gamma-irradiated BCG has no effect on the innate immune system and therefore does not induce trained immunity. This is evident from both the lack of an effect on LPS-induced plasma cytokine levels* in vivo* and similar* ex vivo* innate cytokine responses (TNF-*α*, IL-1*β*, IL-6, and IL-10) against unrelated pathogens five days after vaccination in both groups. Previous epidemiological studies have shown that scar formation after vaccinia or BCG vaccination is associated with improved survival, possibly related to improved resistance against infections [27, 28]. Therefore, we stratified subjects based on scar size, but no effects were found in these analyses either. Furthermore, no effects indicative of trained immunity induction were found at later time points, ranging from 8–10 days to 25–35 days after vaccination.

Our results are different from previous studies that used the live attenuated BCG vaccine [17, 21] instead of the gamma-irradiated BCG. There are several reasons and/or limitations of the present study that might explain these differences. First and foremost, we used gamma-irradiated BCG in the present study because our target treatment population consists of immunoparalyzed septic patients who may be at risk for disseminated mycobacterial infection [22]. We hypothesized that gamma-irradiated BCG would be effective in inducing trained immunity* in vivo* because recent unpublished data of our group showed that gamma-irradiated BCG exerts monocyte training* in vitro*. Furthermore, previous* in vitro* studies showed that monocytes could be trained with live BCG, as well as with the inert NOD2 ligand MDP [17], highlighting that live BCG persistence is not mandatory for inducing trained immunity* in vitro*. Nevertheless, inactivating the vaccine could have reduced or abrogated the “training capacity” of BCG. While live vaccines can replicate and/or disseminate in the host's body and thereby trigger the immune response to a greater extent, inactivated vaccines only activate immune responses locally [30]. Although the scar formation in gamma-irradiated BCG-vaccinated subjects indicates a local immune response, it could be envisioned that possible training effects of gamma-irradiated BCG are much less sustained and widespread and thus less pronounced. Along these lines, it was demonstrated that two and four weeks after vaccination with live BCG, 83 and 50% of individuals still displayed viable BCG at the vaccination site [31], respectively, indicative of a relatively long-lasting “active infectious pool” of bacteria (or their products) and/or cytokines that trigger a variety of responses. This is likely not relevant for the* in vitro* situation, where cells are continuously exposed to bacteria and/or their products irrespective of whether they are alive or inactivated. Also, others have shown that viable bacteria elicit more potent immune responses compared to killed bacteria, due to recognition of so-called “vita-PAMPs” such as prokaryotic mRNA by innate immune cells [32]. The absent effects of gamma-irradiated BCG on* ex vivo* cytokine responses to stimulation with* M. tuberculosis* further substantiate the hypothesis that gamma-irradiation results in functional inactivation of BCG, resulting in abrogation not only of trained immunity but also of “classic” specific protection against* M. tuberculosis.*

Secondly, the timing of the interventions in our study might have precluded effects of gamma-irradiated BCG. We chose to assess* in vivo* and* ex vivo* responses already five days after vaccination, in order to assess potential short-term effects that may be most relevant during sepsis. We hypothesized that this period would be sufficient to induce trained immunity based on the fact that* in vitro* training by BCG only takes one day and that nonspecific beneficial effects of BCG vaccination in neonates were already apparent within 3 days [15]. Nevertheless, in previous studies enhancing effects of BCG on leukocytes were found 2 weeks, 3 months, and one year after vaccination [17, 21]. No earlier time points were assessed in these earlier studies.

Thirdly, we only included young male volunteers in this study. There are considerable differences in the cytokine response to LPS between males and females [33]. This is likely influenced by menstrual cycle-related hormonal variations that can affect the immune response. Because we wanted our study population to be as homogenous as possible, we therefore included only males, analogous to virtually all of our previous endotoxemia studies. This might have biased our results, because the majority of the studies on nonspecific effect of the BCG vaccine point to important sex-differential nonspecific effects and often the most pronounced effects were observed among females [12, 34, 35]. Nonspecific effects may also vary with age; nevertheless, live BCG exerted profound effects in a similarly aged study population [17, 21].

Fourthly, possible training effects of gamma-irradiated BCG on monocytes in the long term might have been obscured by the LPS administration five days after vaccination. BCG-induced trained immunity has been shown to be mediated through epigenetic reprogramming of monocytes [17]. Interestingly, exposure to LPS results in opposite epigenetic changes in monocytes and/or macrophages [20]. Therefore, possible training effects induced by gamma-irradiated BCG might have been nullified by the LPS administration.

Fifthly, the human endotoxemia model employed in this study is relatively mild and does not replicate the severe sepsis-induced immunoparalysis observed in actual patients. Therefore, we cannot exclude the possibility that gamma-irradiated BCG exerts immunomodulatory effects in a true model of immunoparalysis or in immunoparalyzed septic patients, although this appears unlikely based on the complete absence of effects in the present study.

Finally, our study is limited by the fact that, apart from medical history, we did not screen our subjects for previous exposure to* Mycobacterium tuberculosis*. We chose not to perform a tuberculin skin test since this could trigger trained immunity effects on its own which would confound our study results. However, the infectious pressure of tuberculosis in Netherlands is very low [36], and this possibility is unlikely to explain the absent effects of gamma-irradiated BCG.

In view of the points raised above, a study using live BCG and possibly other timing of the interventions could be considered. While such a study would be warranted to elucidate the mechanisms behind the important nonspecific beneficial effects of BCG vaccination in neonates [9–15], it may be less relevant with regard to sepsis patients, in which the use of live BCG vaccine would be associated with too high risks.

## 5. Conclusions

Gamma-irradiated BCG does not modulate the* in vivo* innate immune response in adult male volunteers five days after vaccination. Furthermore, vaccination did not induce trained immunity* ex vivo.* Therefore, gamma-irradiated BCG is unlikely to represent a viable treatment option to restore immunocompetence in patients with sepsis-induced immunoparalysis.

## Supplementary Material

The Supplementary Material consists of Supplementary Figures 1, 2, and 3. Supplementary Figure 1 depicts Area under curve (AUC) of plasma cytokine concentrations within the BCG-vaccinated group stratified according to scar size. Supplementary Figure 2 depicts production of IL-6 and IL-10 by peripheral blood mononuclear cells stimulated ex vivo with various specific and unrelated pathogens or stimuli. Supplementary Figure 3 depicts all ex vivo cytokine data already shown in Figure 4 and Supplementary Figure 2, but in absolute values instead of fold change compared with baseline.
Supplementary Figure 1. Area under curve (AUC) of plasma concentrations of pro-inflammatory cytokines TNF-a, IL-6, IL-8, and MCP-1, and anti-inflammatory cytokines IL-10 and IL-1RA in subjects vaccinated with gamma-irradiated BCG stratified according to vaccination scar size (=5 mm or >5 mm, n=5 per group). Data are presented as median ± interquartile range of the respective cytokines. P values calculated using Mann-Whitney U-tests.
Supplementary Figure 2. Production of IL-6 and IL-10 by peripheral blood mononuclear cells stimulated ex vivo with Mycobacterium tuberculosis (MTB), LPS, Staphylococcus aureus (SA), and Candica albicans (CA) of subjects vaccinated with gamma-irradiated BCG or placebo. SA- and CA-induced IL-10 production was absent in virtually all subjects and was therefore not analyzed. Data expressed as median and interquartile range of the fold change compared with day 1 (before vaccination) (n=10 per group). p-values calculated using repeated measures two-way analysis of variance (ANOVA, time and interaction terms) on log transformed data. Day 6 was the endotoxemia experiment day.
Supplementary Figure 3. Production of IFN-gamma, TNF-a, IL-1ß, IL-6, and IL-10 by peripheral blood mononuclear cells stimulated ex vivo with Mycobacterium tuberculosis (MTB), LPS, Staphylococcus aureus (SA), and Candica albicans (CA) of subjects vaccinated with gamma-irradiated BCG or placebo. Data expressed as median and interquartile range (n=10 per group). Day 6 was the endotoxemia experiment day. For statistical analyses, see Figures 4 and supplementary Figure 2, which depict the same data expressed as fold change compared with day 1.

## Figures and Tables

**Table 1:** Baseline characteristics.

	Placebo (*n* = 10)	Gamma-irradiated BCG (*n* = 10)	*P* value
Age, y	20.0 (19.0–24.3)	20.5 (19.8–22.0)	0.82
Height, cm	183.0 (178.3–193.0)	183.5 (178.8–189.8)	0.91
Weight, kg	80.8 (70.3–83.5)	78.9 (76.4–93.4)	0.58
BMI, kg/m^2^	22.7 (21.5–24.7)	24.2 (23.1–25.8)	0.09
Heart rate, bpm	66.0 (59.0–76.3)	70.0 (64.8–82.0)	0.30
MAP, mmHg	97.0 (95.0–103.8)	93.0 (89.5–99.3)	0.14

BCG: Bacille Calmette-Guérin; BMI: body mass index; MAP: mean arterial pressure.

Data are represented as median (interquartile range). *P* values calculated using Mann-Whitney *U* tests.

## References

[1] Boomer J. S., To K., Chang K. C. (2011). Immunosuppression in patients who die of sepsis and multiple organ failure. *The Journal of the American Medical Association*.

[2] Hamers L., Kox M., Pickkers P. Sepsis-induced immunoparalysis: mechanisms, markers, and treatment options.

[3] Leentjens J., Kox M., van der Hoeven J. G., Netea M. G., Pickkers P. (2013). Immunotherapy for the adjunctive treatment of sepsis: from immunosuppression to immunostimulation time for a paradigm change?. *The American Journal of Respiratory and Critical Care Medicine*.

[4] Tulzo Y. L., Pangault C., Amiot L. (2004). Monocyte human leukocyte antigen-DR transcriptional downregulation by cortisol during septic shock. *American Journal of Respiratory and Critical Care Medicine*.

[5] Otto G. P., Sossdorf M., Claus R. A. (2011). The late phase of sepsis is characterized by an increased microbiological burden and death rate. *Critical Care*.

[6] Angus D. C., van der Poll T. (2013). Severe sepsis and septic shock. *The New England Journal of Medicine*.

[7] Meisel C., Schefold J. C., Pschowski R. (2009). Granulocyte-macrophage colony-stimulating factor to reverse sepsis-associated immunosuppression: a double-blind, randomized, placebo-controlled multicenter trial. *American Journal of Respiratory and Critical Care Medicine*.

[8] Colditz G. A., Brewer T. F., Berkey C. S. (1994). Efficacy of BCG vaccine in the prevention of tuberculosis: Meta-analysis of the published literature. *The Journal of the American Medical Association*.

[9] Levine M. I., Sackett M. F. (1946). Results of BCG immunization in New York City. *The American Review of Tuberculosis*.

[10] Rosenthal S. R., Loewinsohn E., Graham M. L. (1961). BCG vaccination in tuberculous households. *The American Review of Respiratory Disease*.

[11] Breiman R. F., Streatfield P. K., Phelan M., Shifa N., Rashid M., Yunus M. (2004). Effect of infant immunisation on childhood mortality in rural Bangladesh: analysis of health and demographic surveillance data. *The Lancet*.

[12] Roth A., Jensen H., Garly M.-L. (2004). Low birth weight infants and Calmette-Guérin bacillus vaccination at birth: community study from Guinea-Bissau. *Pediatric Infectious Disease Journal*.

[13] Kristensen I., Aaby P., Jensen H. (2000). Routine vaccinations and child survival: follow up study in Guinea-Bissau, West Africa. *British Medical Journal*.

[14] Aaby P., Roth A., Ravn H. (2011). Randomized trial of BCG vaccination at birth to low-birth-weight children: beneficial nonspecific effects in the neonatal period?. *The Journal of Infectious Diseases*.

[15] Biering-Sørensen S., Aaby P., Napirna B. M. (2012). Small randomized trial among low-birth-weight children receiving bacillus Calmette-Guéerin vaccination at first health center contact. *Pediatric Infectious Disease Journal*.

[16] Flanagan K. L., van Crevel R., Curtis N., Shann F., Levy O. (2013). Heterologous (‘nonspecific’) and sex-differential effects of vaccines: epidemiology, clinical trials, and emerging immunologic mechanisms. *Clinical Infectious Diseases*.

[17] Kleinnijenhuis J., Quintin J., Preijers F. (2012). Bacille Calmette-Guérin induces NOD2-dependent nonspecific protection from reinfection via epigenetic reprogramming of monocytes. *Proceedings of the National Academy of Sciences of the United States of America*.

[18] Netea M. G., van Crevel R. (2014). BCG-induced protection: effects on innate immune memory. *Seminars in Immunology*.

[19] Quintin J., Cheng, S.-C., van der Meer J. W. M., Netea M. G. (2014). Innate immune memory: towards a better understanding of host defense mechanisms. *Current Opinion in Immunology*.

[20] Saeed S., Quintin J., Kerstens H. H. D. (2014). Epigenetic programming of monocyte-to-macrophage differentiation and trained innate immunity. *Science*.

[21] Kleinnijenhuis J., Quintin J., Preijers F. (2014). Long-lasting effects of BCG vaccination on both heterologous Th1/Th17 responses and innate trained immunity. *Journal of Innate Immunity*.

[22] Talbot E. A., Perkins M. D., Suva S. F. M., Frothingham R. (1997). Disseminated bacille Calmette-Guerin disease after vaccination: case report and review. *Clinical Infectious Diseases*.

[23] Dorresteijn M. J., Draisma A., van der Hoeven J. G., Pickkers P. (2010). Lipopolysaccharide-stimulated whole blood cytokine production does not predict the inflammatory response in human endotoxemia. *Innate Immunity*.

[24] Kox M., De Kleijn S., Pompe J. C. (2011). Differential ex vivo and in vivo endotoxin tolerance kinetics following human endotoxemia. *Critical Care Medicine*.

[25] Bahador M., Cross A. S. (2007). Review: from therapy to experimental model: a hundred years of endotoxin administration to human subjects. *Journal of Endotoxin Research*.

[26] Kox M., van Eijk L. T., Zwaag J. (2014). Voluntary activation of the sympathetic nervous system and attenuation of the innate immune response in humans. *Proceedings of the National Academy of Sciences of the United States of America*.

[27] Jensen M. L., Dave S., van der Loeff M. S. (2006). Vaccinia scars associated with improved survival among adults in rural Guinea-Bissau. *PLoS ONE*.

[28] Roth A., Gustafson P., Nhaga A. (2005). BCG vaccination scar associated with better childhood survival in Guinea-Bissau. *International Journal of Epidemiology*.

[29] de Vos A. F., Pater J. M., van den Pangaart P. S., de Kruif M. D., van 't Veer C., van der Poll T. (2009). In vivo lipopolysaccharide exposure of human blood leukocytes induces cross-tolerance to multiple TLR ligands. *Journal of Immunology*.

[30] Cox R. J., Brokstad K. A., Ogra P. (2004). Influenza virus: immunity and vaccination strategies. Comparison of the immune response to inactivated and live, attenuated influenza vaccines. *Scandinavian Journal of Immunology*.

[31] Minassian A. M., Satti I., Poulton I. D., Meyer J., Hill A. V. S., McShane H. (2012). A human challenge model for *Mycobacterium tuberculosis* using *Mycobacterium bovis* bacille Calmette-Guérin. *Journal of Infectious Diseases*.

[32] Sander L. E., Davis M. J., Boekschoten M. V. (2011). Detection of prokaryotic mRNA signifies microbial viability and promotes immunity. *Nature*.

[33] van Eijk L. T., Dorresteijn M. J., Smits P., van der Hoeven J. G., Netea M. G., Pickkers P. (2007). Gender differences in the innate immune response and vascular reactivity following the administration of endotoxin to human volunteers. *Critical Care Medicine*.

[34] Stensballe L. G., Nante E., Jensen I. P. (2005). Acute lower respiratory tract infections and respiratory syncytial virus in infants in Guinea-Bissau: a beneficial effect of BCG vaccination for girls: community based case-control study. *Vaccine*.

[35] Aaby P., Jensen H., Rodrigues A. (2004). Divergent female-male mortality ratios associated with different routine vaccinations among female-male twin pairs. *International Journal of Epidemiology*.

[36] Murray C. J., Ortblad K. F., Guinovart C. (2014). Global, regional, and national incidence and mortality for HIV, tuberculosis, and malaria during 1990–2013: a systematic analysis for the Global Burden of Disease Study 2013. *The Lancet*.

