# Supplementary figures and images for: Gamma-Irradiated Bacille Calmette-Guérin Vaccination Does Not Modulate the Innate Immune Response during Experimental Human Endotoxemia in Adult Males
Source: J Immunol Res. 2015 Mar 26;2015:261864. doi: 10.1155/2015/261864 (PMC4391613; doi:10.1155/2015/261864)

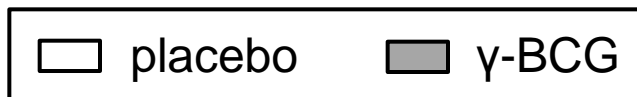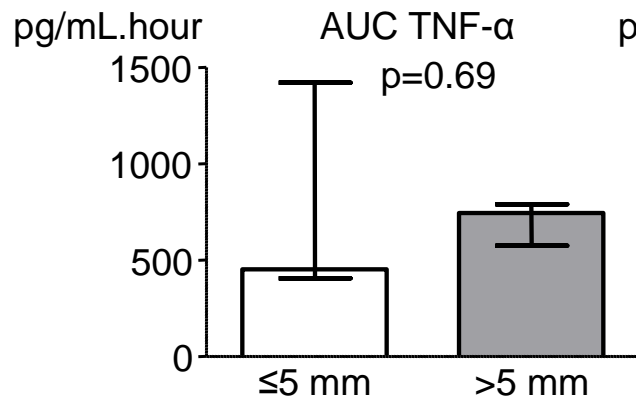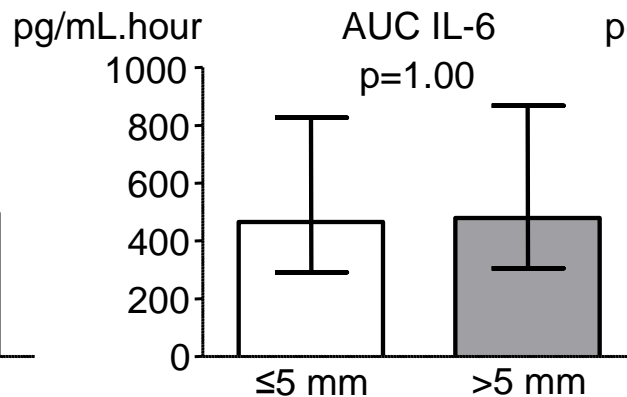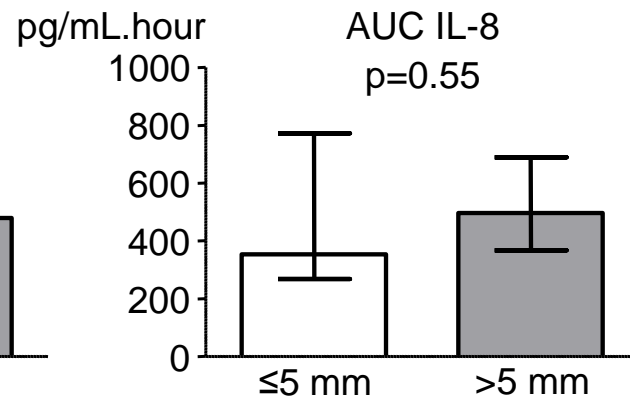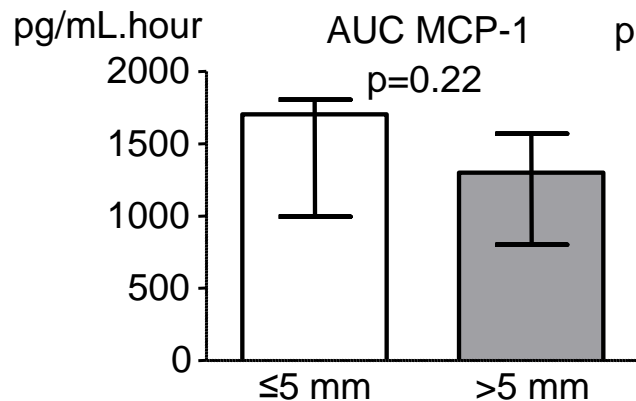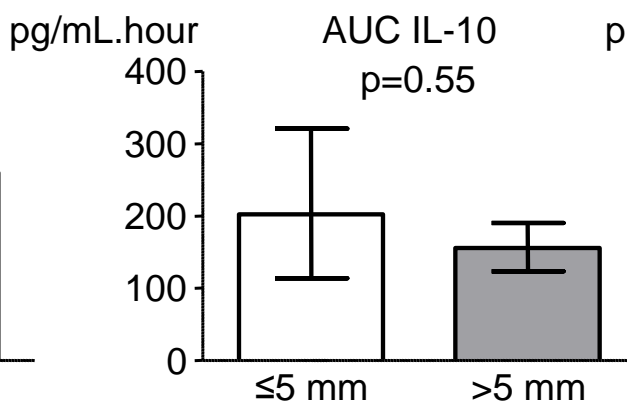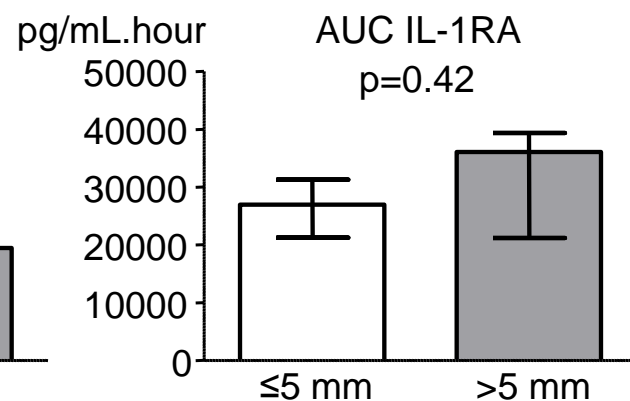

Supplement: Supplementary file 1 — The Supplementary Material consists of Supplementary Figures 1, 2, and 3. Supplementary Figure 1 depicts Area under curve (AUC) of plasma cytokine concentrations within the BCG-vaccinated group stratified according to scar size. Supplementary Figure 2 depicts production of IL-6 and IL-10 by peripheral blood mononuclear cells stimulated ex vivo with various specific and unrelated pathogens or stimuli. Supplementary Figure 3 depicts all ex vivo cytokine data already shown in Figure 4 and Supplementary Figure 2, but in absolute values instead of fold change compared with baseline. Supplementary Figure 1. Area under curve (AUC) of plasma concentrations of pro-inflammatory cytokines TNF-a, IL-6, IL-8, and MCP-1, and anti-inflammatory cytokines IL-10 and IL-1RA in subjects vaccinated with gamma-irradiated BCG stratified according to vaccination scar size (=5 mm or >5 mm, n=5 per group). Data are presented as median ± interquartile range of the respective cytokines. P values calculated using Mann-Whitney U-tests. Supplementary Figure 2. Production of IL-6 and IL-10 by peripheral blood mononuclear cells stimulated ex vivo with Mycobacterium tuberculosis (MTB), LPS, Staphylococcus aureus (SA), and Candica albicans (CA) of subjects vaccinated with gamma-irradiated BCG or placebo. SA- and CA-induced IL-10 production was absent in virtually all subjects and was therefore not analyzed. Data expressed as median and interquartile range of the fold change compared with day 1 (before vaccination) (n=10 per group). p-values calculated using repeated measures two-way analysis of variance (ANOVA, time and interaction terms) on log transformed data. Day 6 was the endotoxemia experiment day. Supplementary Figure 3. Production of IFN-gamma, TNF-a, IL-1ß, IL-6, and IL-10 by peripheral blood mononuclear cells stimulated ex vivo with Mycobacterium tuberculosis (MTB), LPS, Staphylococcus aureus (SA), and Candica albicans (CA) of subjects vaccinated with gamma-irradiated BCG or placebo. Data e [file 261864.f1.pdf]

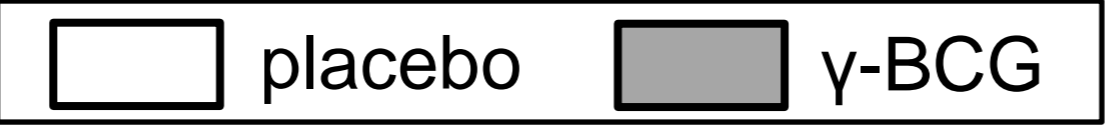

*M. tuberculosis*

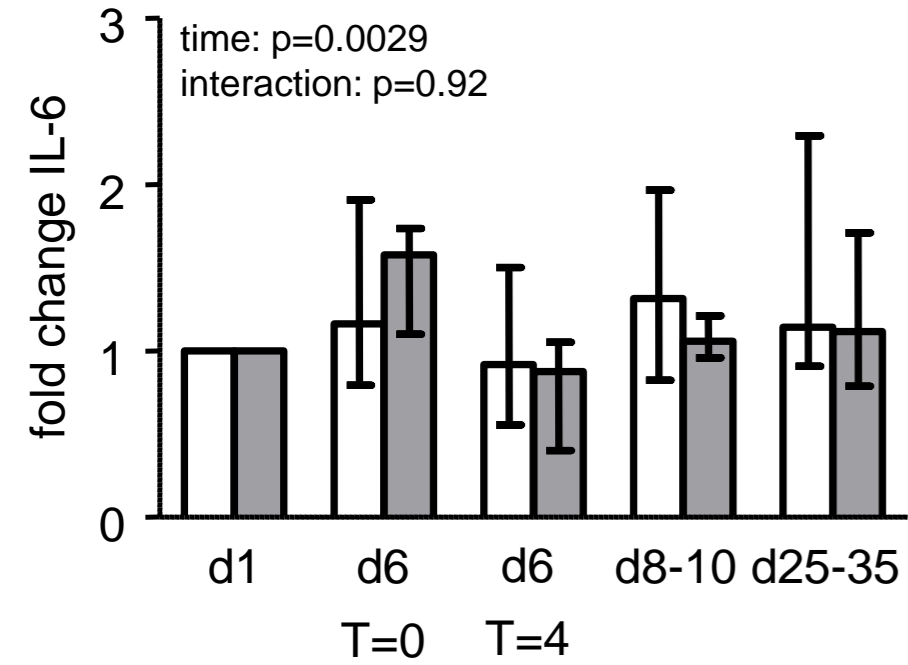

LPS

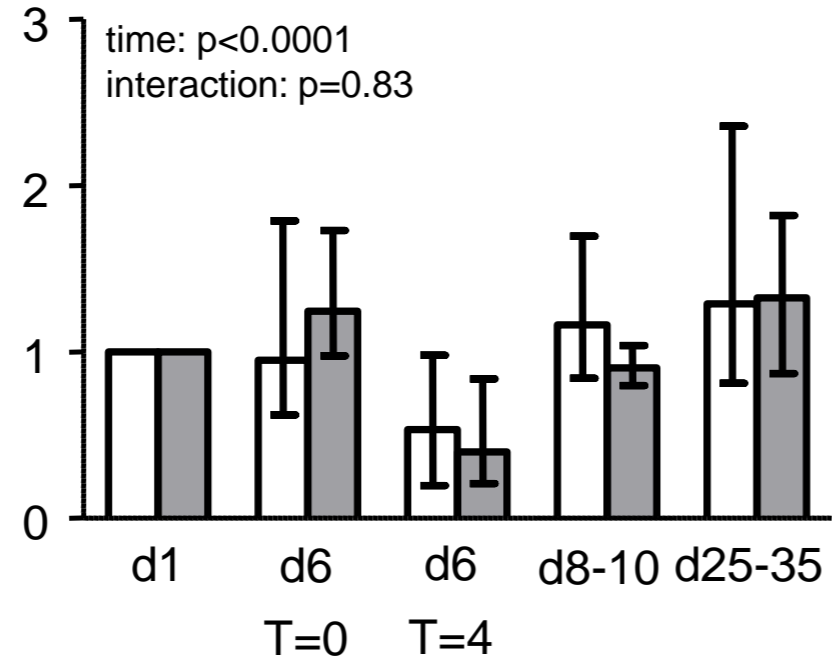

*S. aureus*

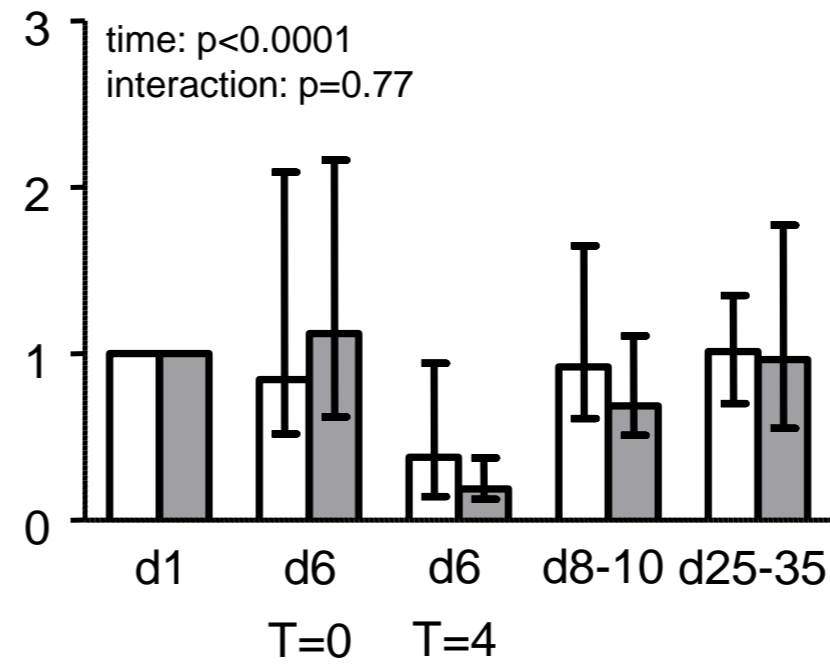

*C. albicans*

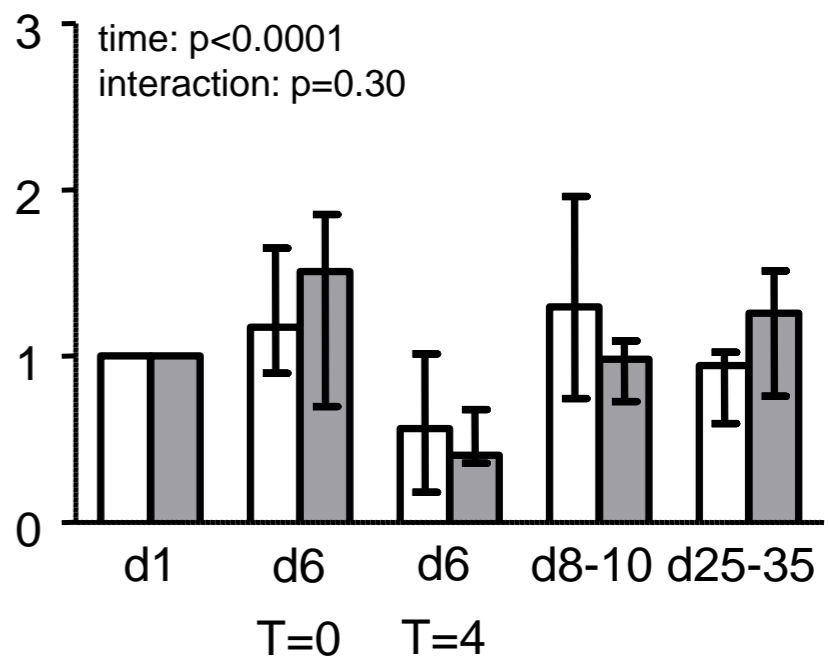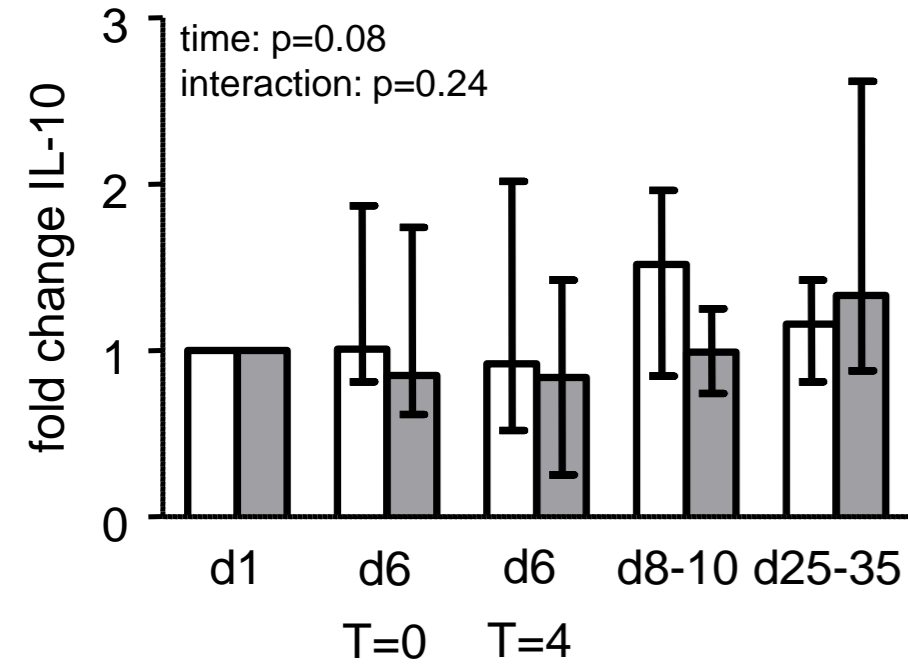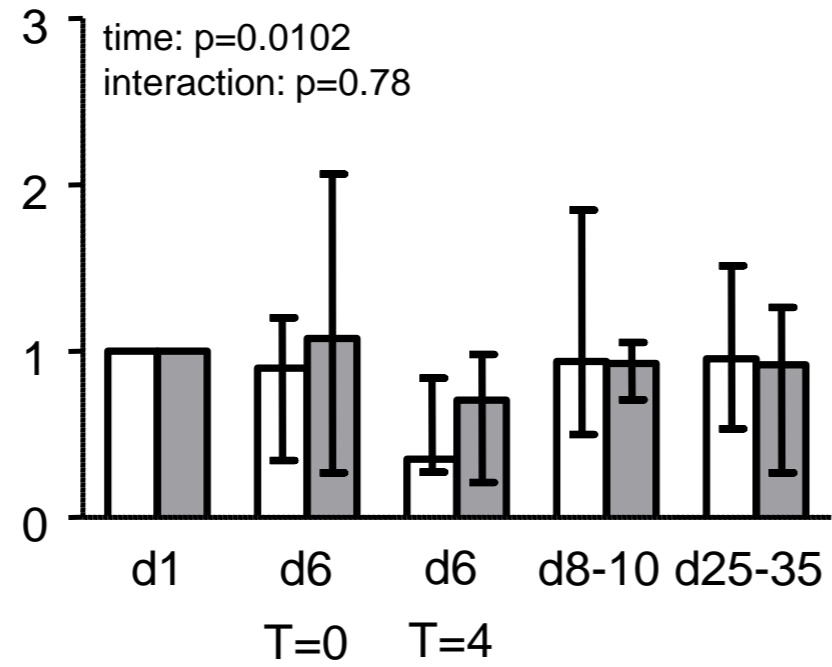

Supplement: Supplementary file 2 [file 261864.f2.pdf]

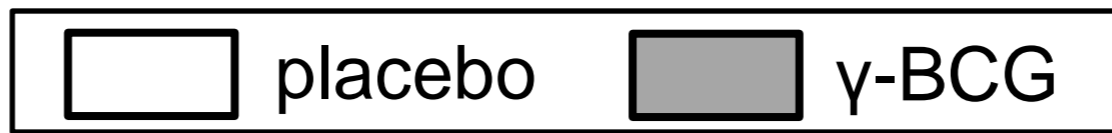

*M. tuberculosis*

LPS

*S. aureus*

*C. albicans*

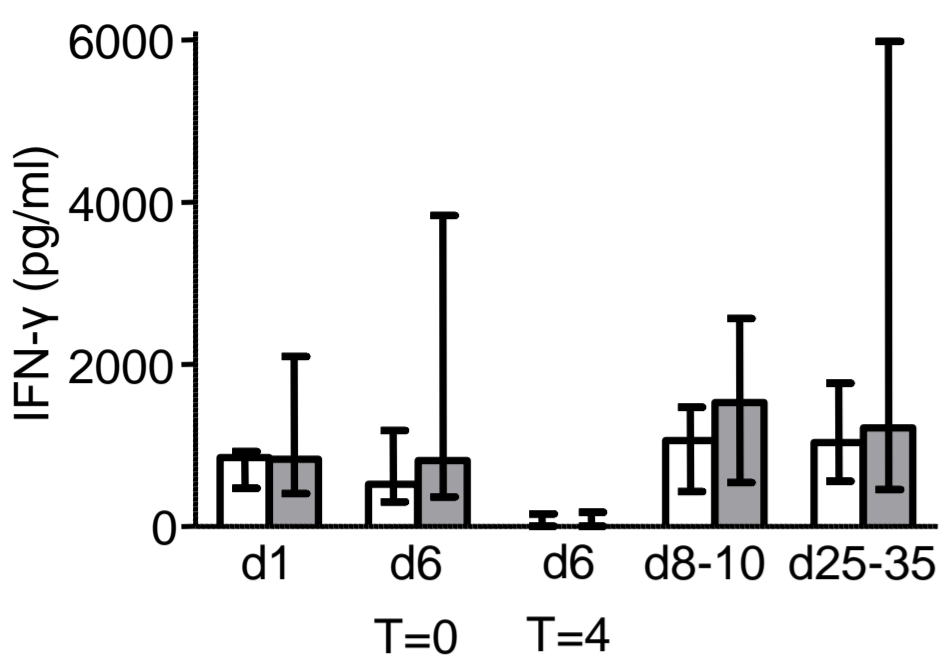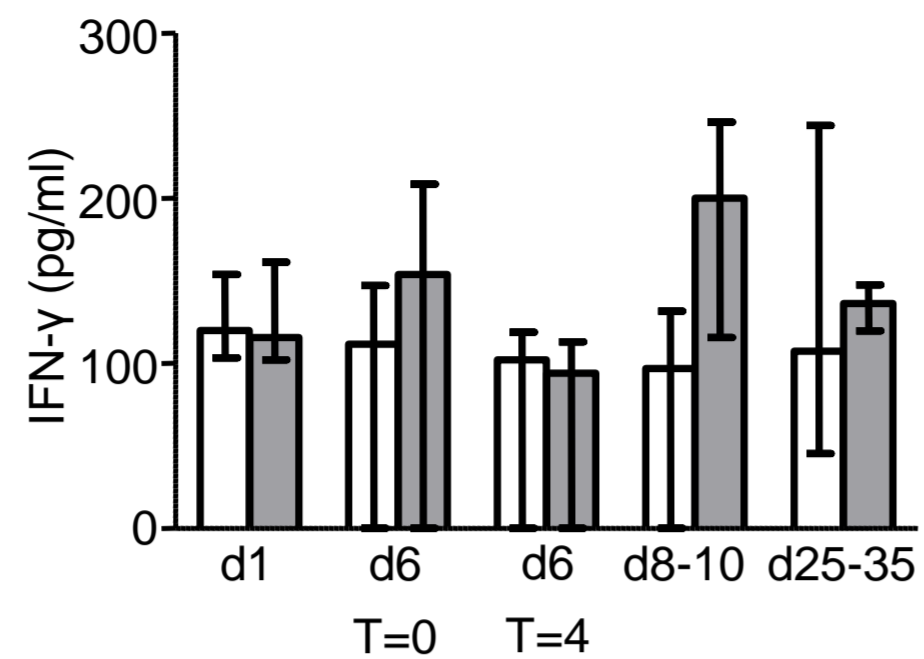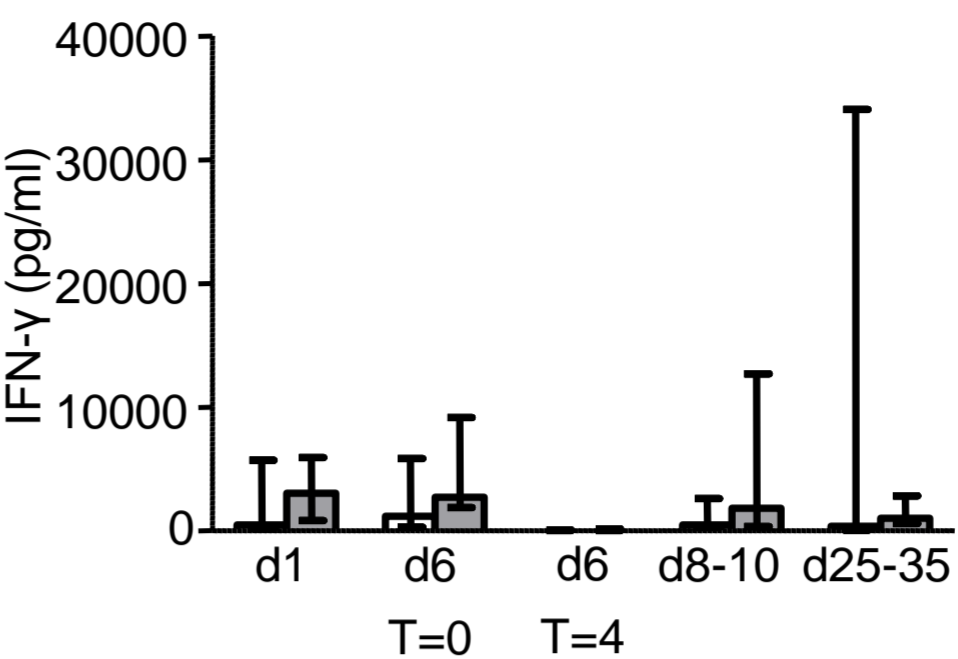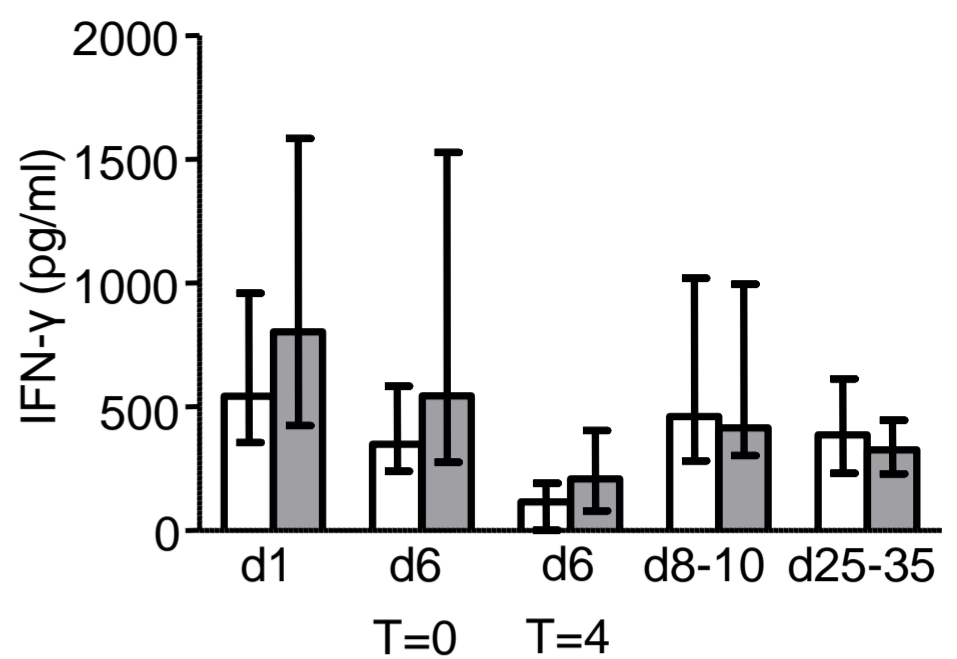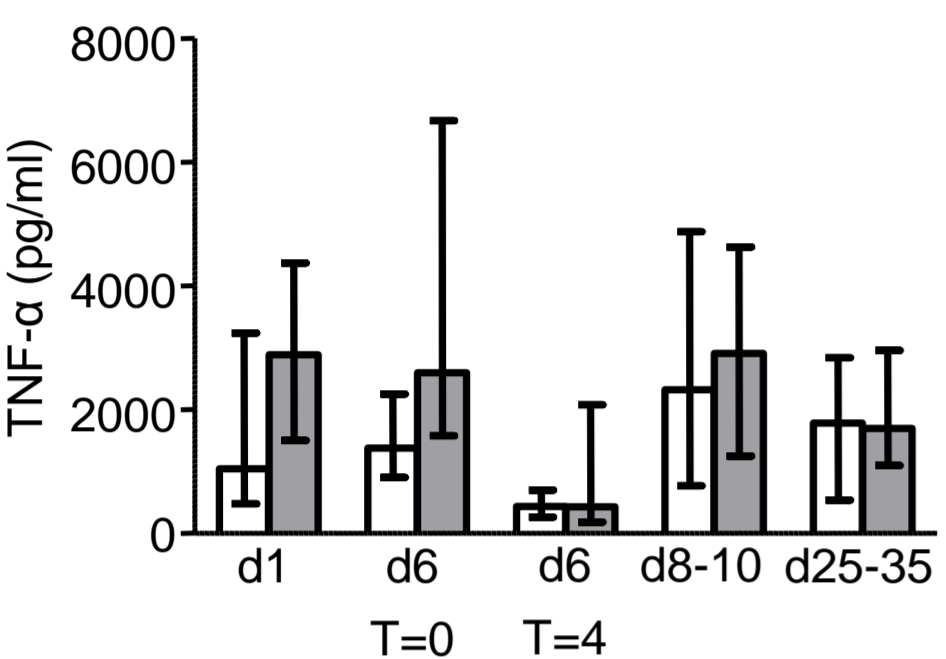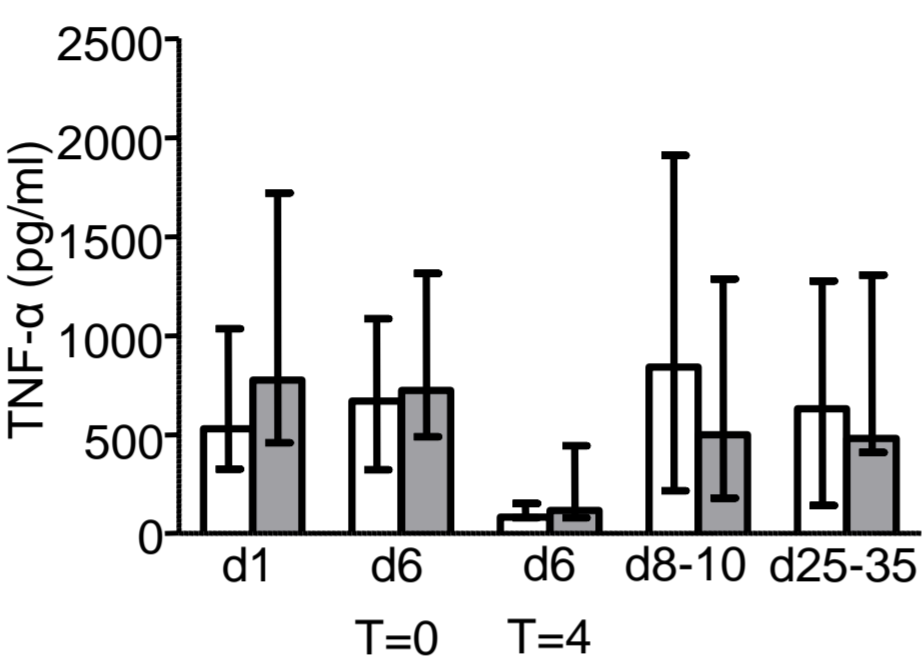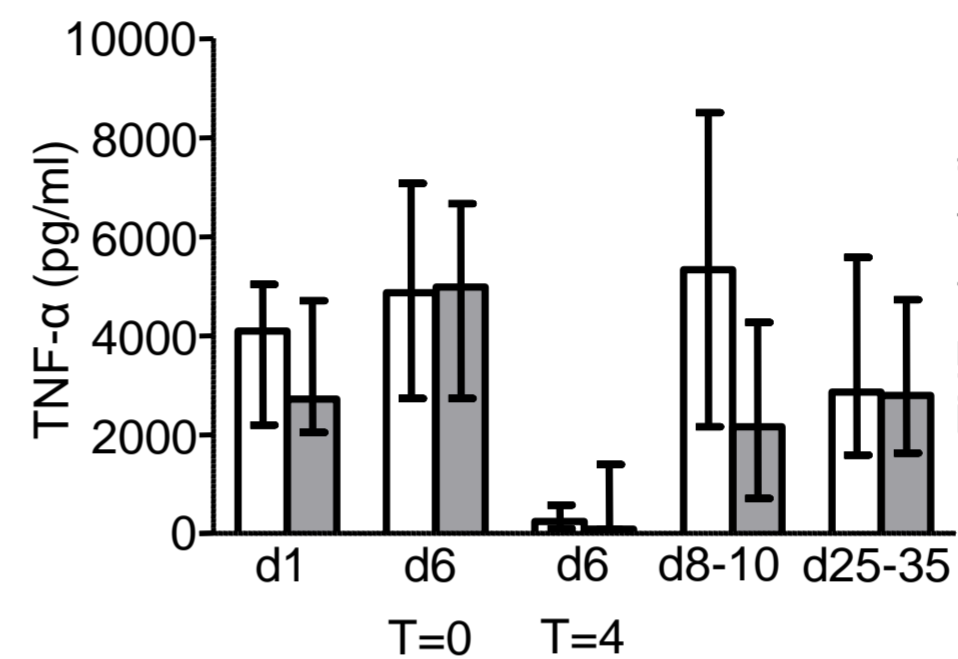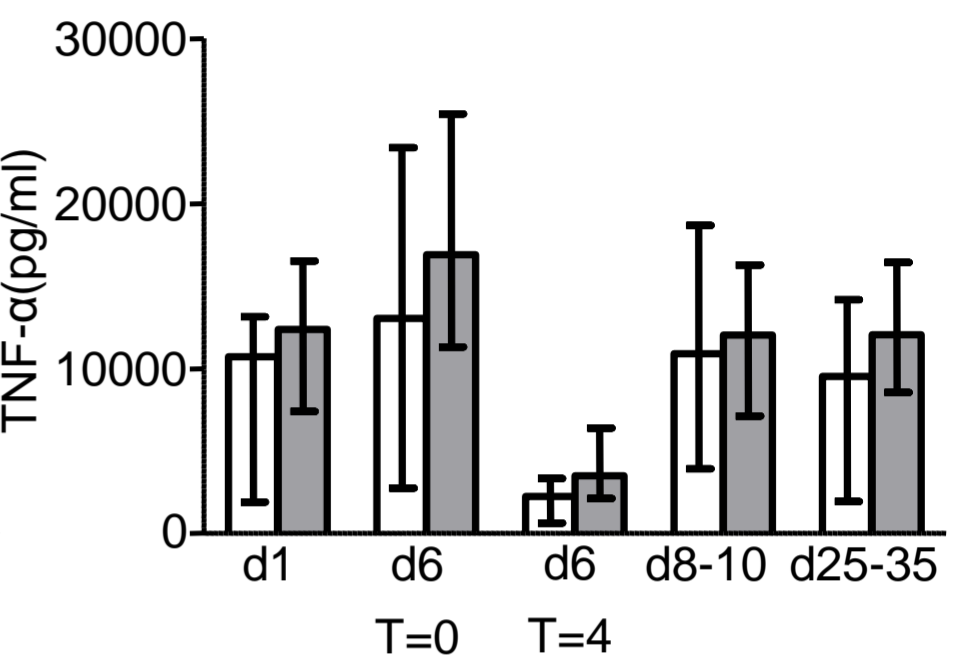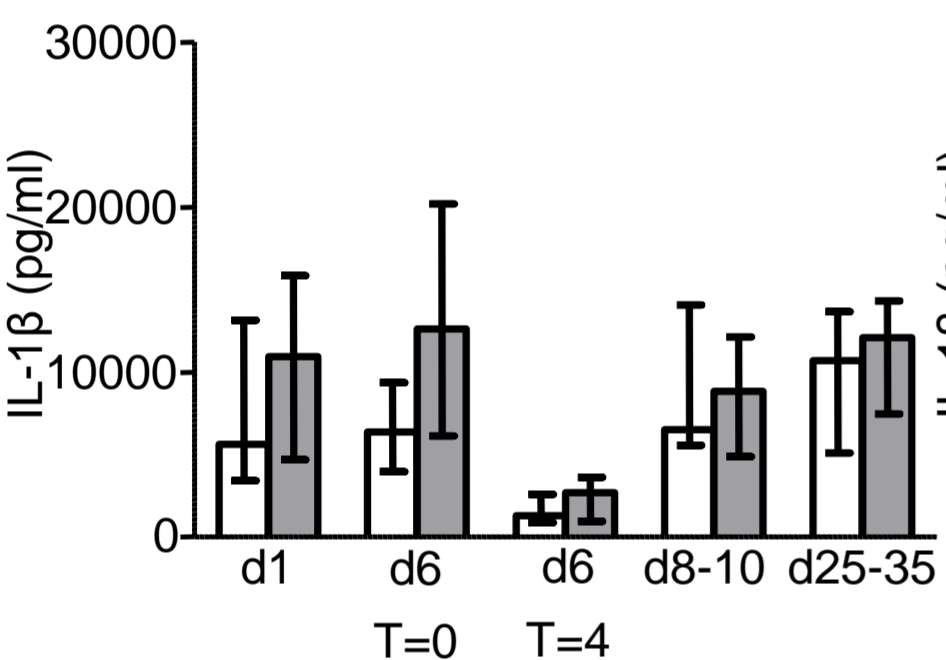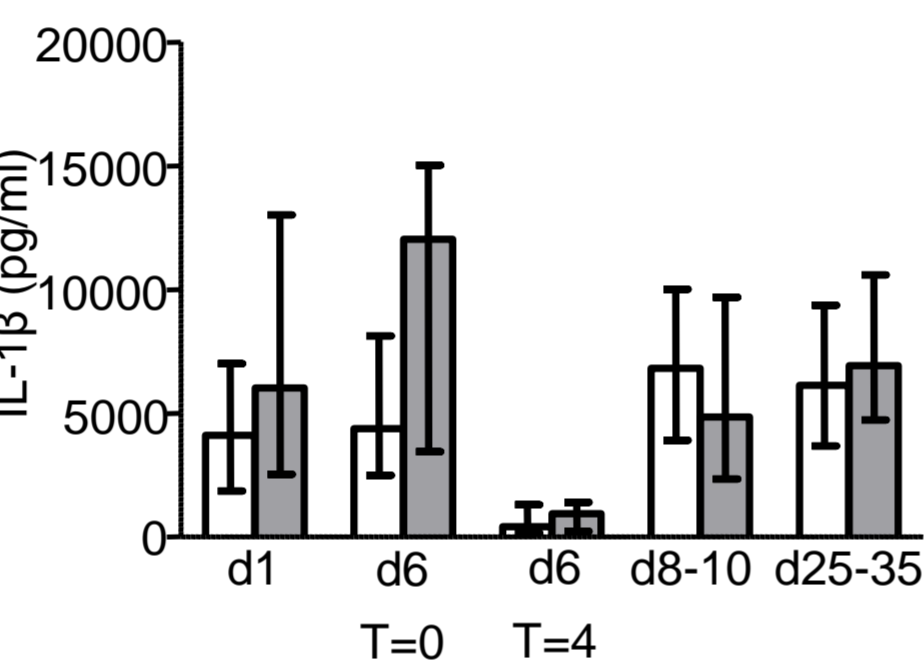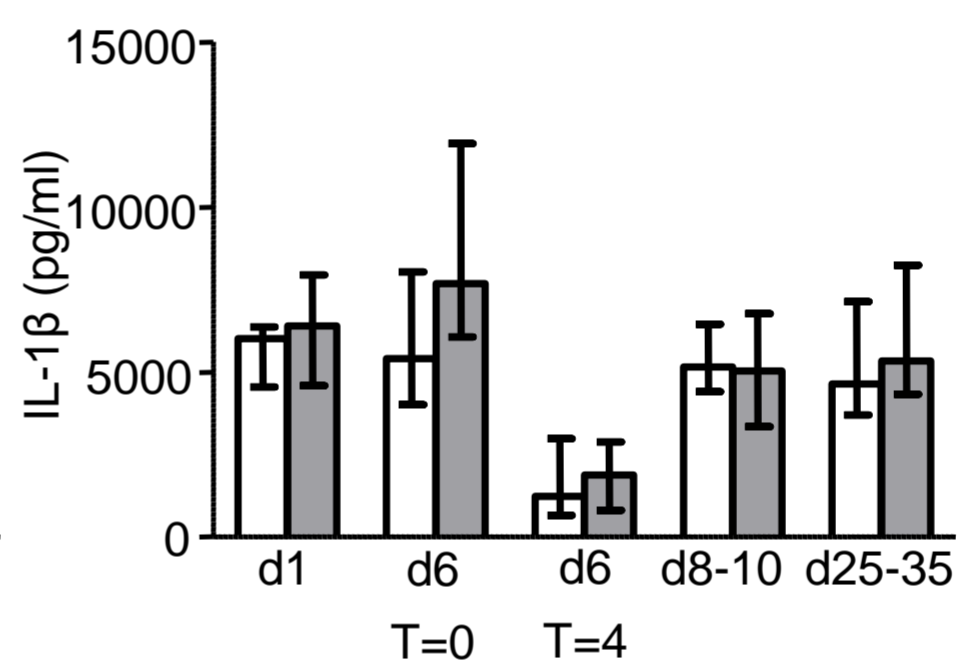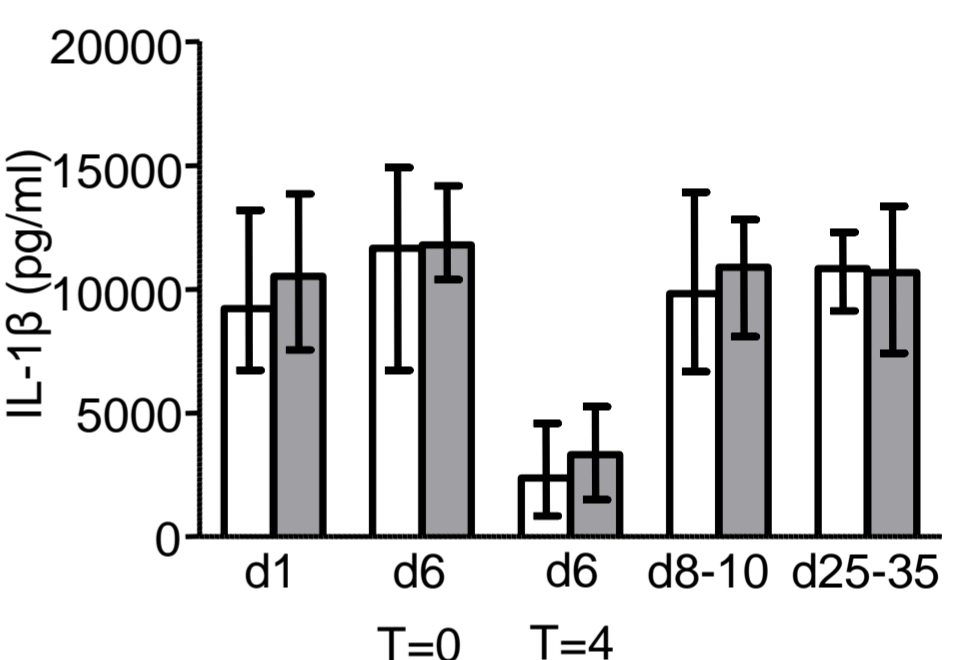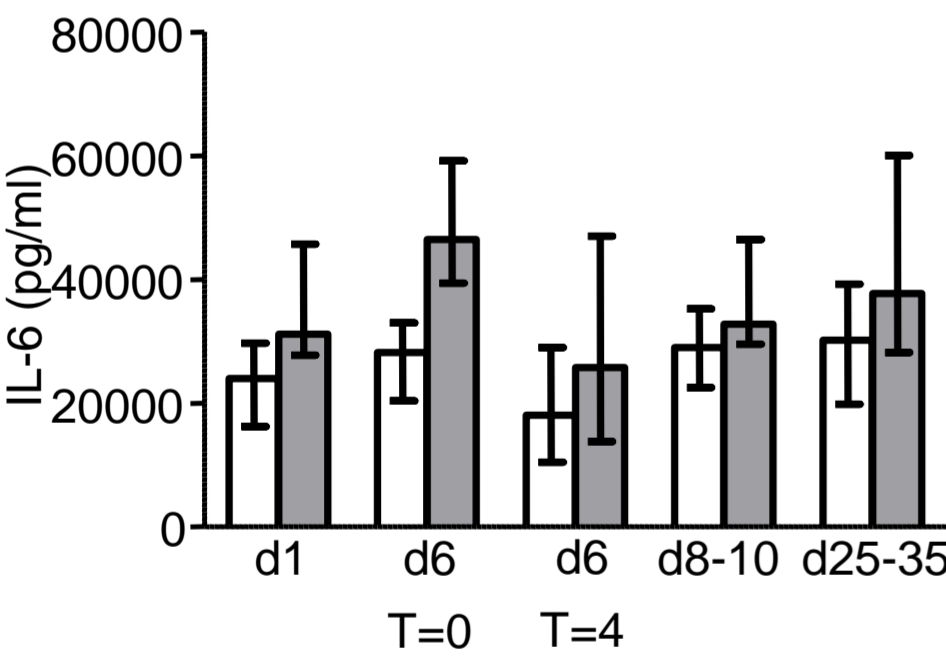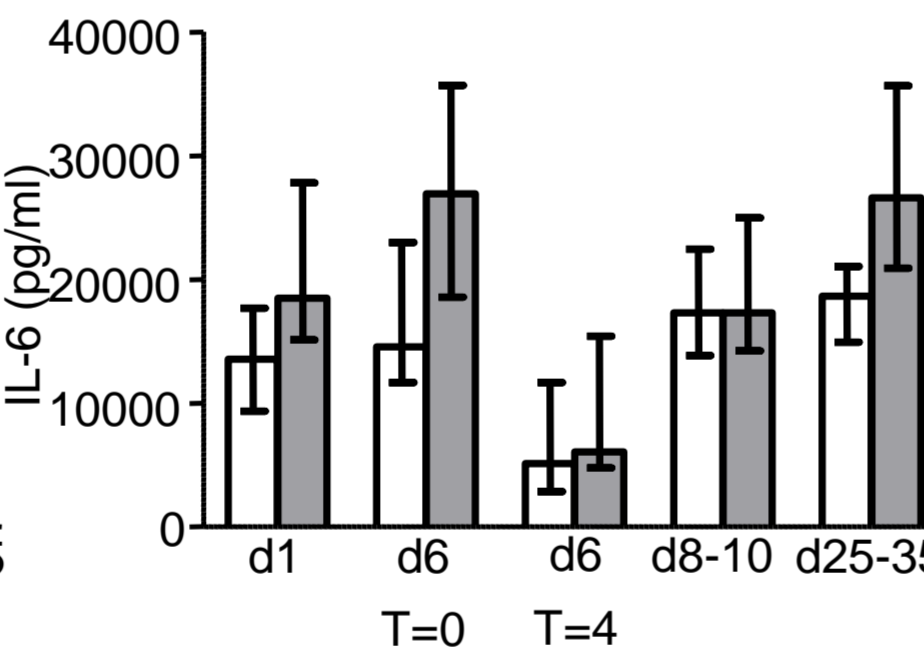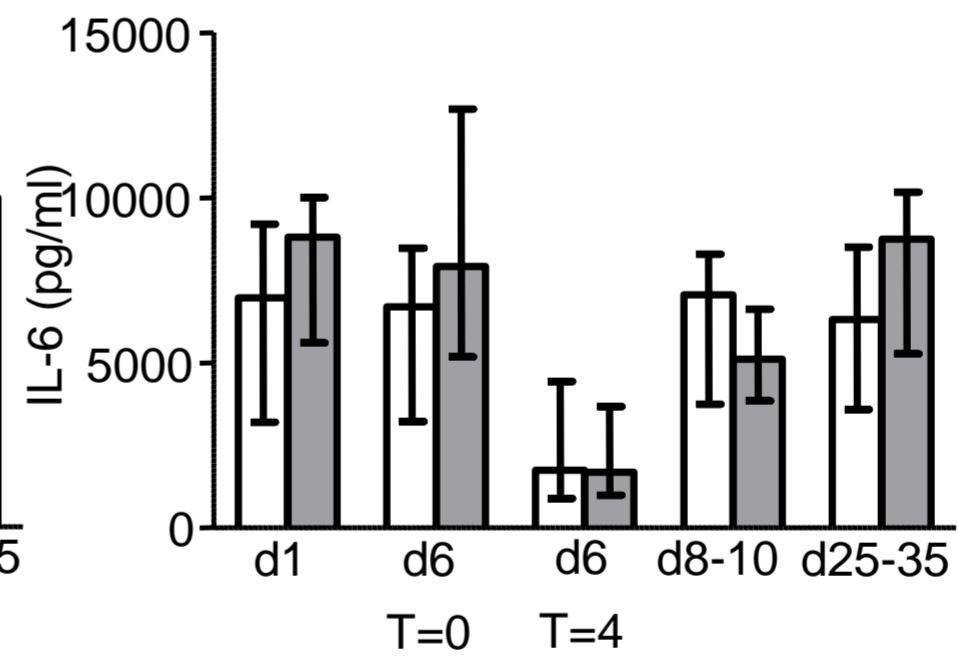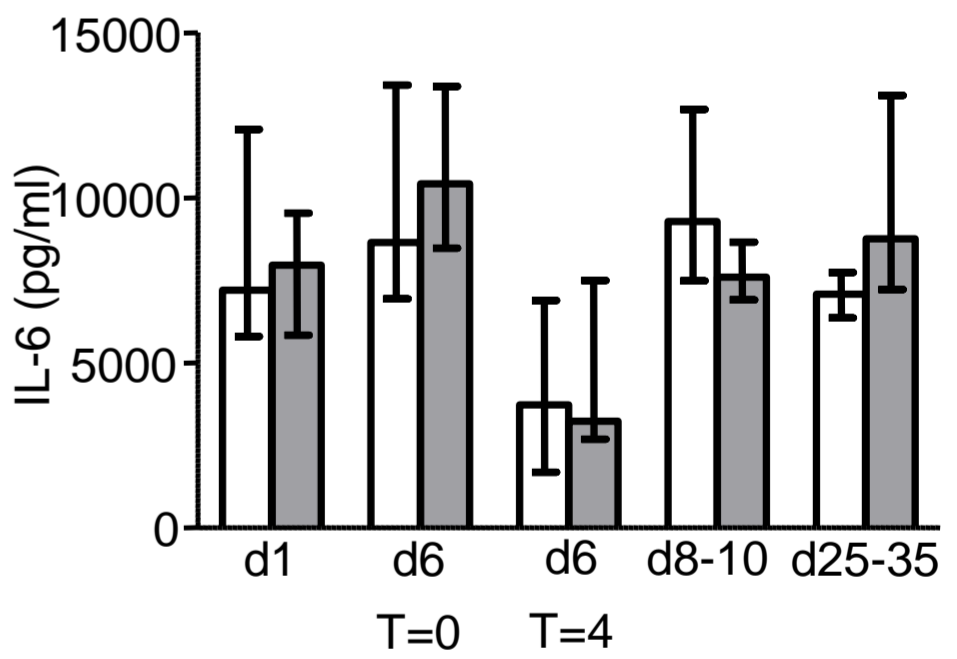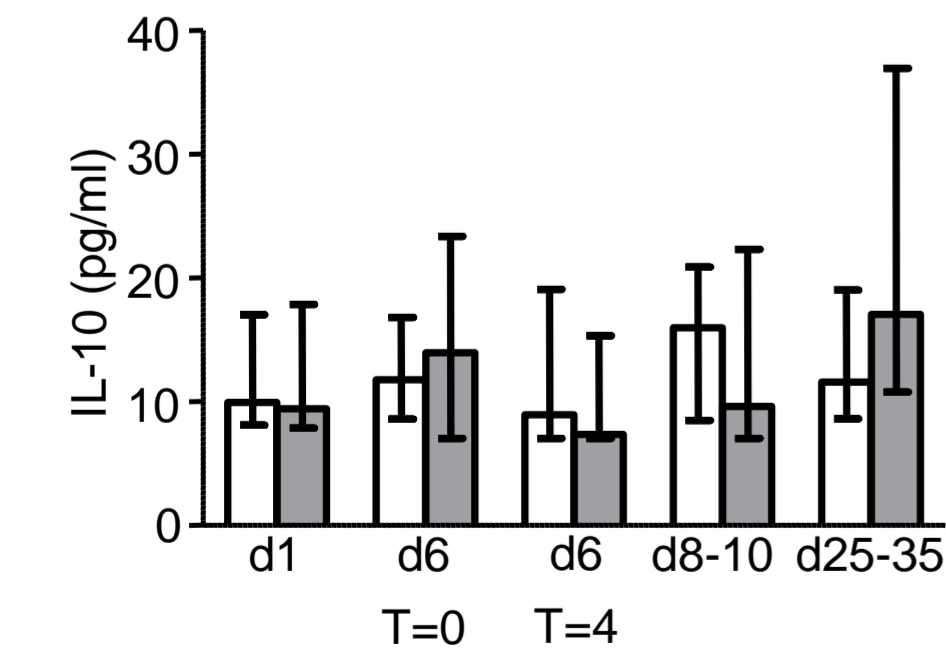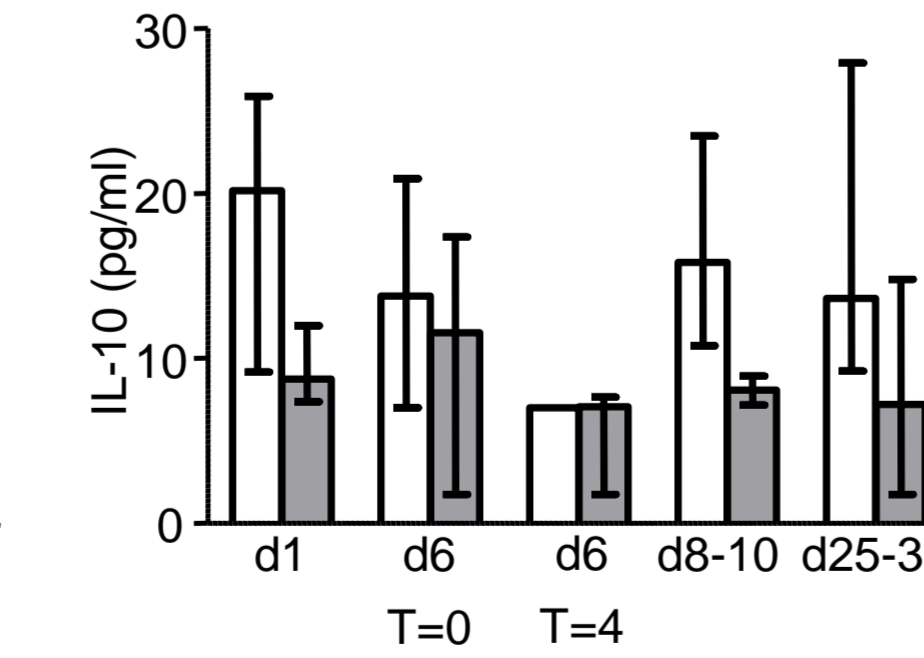

Supplement: Supplementary file 3 [file 261864.f3.pdf]
